# Brain adaptation following various unilateral vocal fold paralysis treatments: A magnetic resonance imaging based longitudinal case series

**DOI:** 10.3389/fnins.2022.947390

**Published:** 2022-10-05

**Authors:** Marie Dedry, Laurence Dricot, Vinciane Van Parys, Donatienne Boucquey, Nicolas Delinte, Julie van Lith-Bijl, Arnaud Szmalec, Youri Maryn, Gauthier Desuter

**Affiliations:** ^1^Psychological Sciences Research Institute, Université catholique de Louvain, Louvain-la-Neuve, Belgium; ^2^Institute of Neuroscience, Université catholique de Louvain, Brussels, Belgium; ^3^Neuromuscular Reference Center, Cliniques Universitaires Saint-Luc, Université catholique de Louvain, Brussels, Belgium; ^4^Otolaryngology, Head and Neck Surgery Department, Voice and Swallowing Clinic, Cliniques Universitaires Saint-Luc, Université catholique de Louvain, Brussels, Belgium; ^5^Institute of Information and Communication Technologies, Electronics and Applied Mathematics (ICTM), Université catholique de Louvain, Louvain-la-Neuve, Belgium; ^6^ENT Department, Flevoziekenhuis, Almere, Netherlands; ^7^Department of Experimental Psychology, Faculty of Psychology and Educational Science, Ghent University, Ghent, Belgium; ^8^Department of Otorhinolaryngology and Head and Neck Surgery, European Institute for ORL-HNS, Sint-Augustinus (GZA), Antwerp, Belgium; ^9^Department of Rehabilitation Sciences and Physiotherapy, Faculty of Medicine and Health Sciences, Ghent University, Ghent, Belgium; ^10^Faculty of Education, Health and Social Work, University College Ghent, Ghent, Belgium; ^11^Phonanium, Lokeren, Belgium

**Keywords:** unilateral vocal fold paralysis, UVFP, early intervention, fMRI, sustained phonation, brain plasticity, nerve recovery, voice recovery

## Abstract

**Aim:**

Examination of central compensatory mechanisms following peripheral vocal nerve injury and recovery is essential to build knowledge about plasticity of the neural network underlying phonation. The objective of this prospective multiple-cases longitudinal study is to describe brain activity in response to unilateral vocal fold paralysis (UVFP) management and to follow central nervous system adaptation over time in three patients with different nervous and vocal recovery profiles.

**Materials and methods:**

Participants were enrolled within 3 months of the onset of UVFP. Within 1 year of the injury, the first patient did not recover voice or vocal fold mobility despite voice therapy, the second patient recovered voice and mobility in absence of treatment and the third patient recovered voice and vocal fold mobility following an injection augmentation with hyaluronic acid in the paralyzed vocal fold. These different evolutions allowed comparison of individual outcomes according to nervous and vocal recovery. All three patients underwent functional magnetic resonance imaging (fMRI task and resting-state) scans at three (patient 1) or four (patients 2 and 3) time points. The fMRI task included three conditions: a condition of phonation and audition of the sustained [a:] vowel for 3 s, an audition condition of this vowel and a resting condition. Acoustic and aerodynamic measures as well as laryngostroboscopic images and laryngeal electromyographic data were collected.

**Results and conclusion:**

This study highlighted for the first time two key findings. First, hyperactivation during the fMRI phonation task was observed at the first time point following the onset of UVFP and this hyperactivation was related to an increase in resting-state connectivity between previoulsy described phonatory regions of interest. Second, for the patient who received an augmentation injection in the paralyzed vocal fold, we subsequently observed a bilateral activation of the voice-related nuclei in the brainstem. This new observation, along with the fact that for this patient the resting-state connectivity between the voice motor/sensory brainstem nuclei and other brain regions of interest correlated with an aerodynamic measure of voice, support the idea that there is a need to investigate whether the neural recovery process can be enhanced by promoting the restoration of proprioceptive feedback.

## Abbreviations

**Table:** 

LEMG	Laryngeal electromyography
MeAF	Mean air flow
P (1, 2, 3)	Patient (1, 2, 3)
T (1, 2, 3, 4)	Time/Session (1, 2, 3, 4)
PHONATION contrast	[PHONATION_AUDITION condition minus AUDITION condition]
MOBILE map	P2T3 + P2T4 + P3T3 + P3T4 + C1T1 + C1T2 + C1T3 + C1T4
PARALYZED map	P1T1 + P1T2 + P1T3 + P2T1 + P2T2 + P3T1 + P3T2
PARALYZED times (for P2 or P3)	T1 + T2
MOBILE times (for P2 or P3)	T3 + T4

## Introduction

Unilateral vocal fold paralysis (UVFP) results in the majority of cases from peripheral nerve damage in the path of the vagus nerve (CN X). The motor neurons of this nerve relay to the ventrolateral portion of the medulla oblongata of the brainstem at the level of the nucleus ambiguus. The intrinsic muscles of the larynx receive motor innervation *via* the recurrent laryngeal nerve except for the crico-thyroid muscle that is innervated by the superior laryngeal nerve. An injury to the vagus nerve or its recurrent branch can therefore lead to the immobility of the vocal fold on the same side of the injury (Rosen et al., 2016). Sensory afferents from the larynx are transmitted by the superior laryngeal nerve and probably also by sensory anastomoses with the recurrent laryngeal nerve to the nucleus of the solitary tract that is also located in the medulla oblongata (Foote and Thibeault, 2021). According to a recent study (Wang H. W. et al., 2020), paralysis is mainly caused, in decreasing order of prevalence, by surgery (mainly following thyroidectomy), tumors (mainly in the lung), or idiopathic causes. Rarer causes include, in the same order, central nerve damage, cardiovascular disorder, trauma and radiation-related disorder. Vocal impairment following paralysis can be very disabling as the voice is often described as hoarse, breathy and of low intensity or aphonic. Patients may also have respiratory or swallowing complaints (Misono and Merati, 2012). This condition can substantially impair the quality of life with repercussions in familial, social and professional spheres. Patients with UVFP report frustration, isolation, fear and an altered self-identity (Francis et al., 2018).

Regarding the management of the UVFP, a first critical question concerns the timing to intervene. Indeed, following nerve damage, there is systematically an attempt of spontaneous reinnervation. This process can either lead to a recovery of mobility of the vocal fold and thus of the voice, or to an improvement of the voice, without recovery of mobility (through synkinetic reinnervation that prevents atrophy of the paralyzed vocal fold and/or favors in a more medial position of the vocal fold), or it can be unsuccessful. Mau et al. (2017) and Mau (2019) reported that, in 96% of cases, if voice recovery was to occur, it would be before 9 months after nerve damage. After 12 months, this percentage increased to 99%. It was therefore recommended not to perform definitive surgical modification (such as medialization laryngoplasty or laryngeal reinnervation) before this time, unless the prognosis for recovery, as assessed with laryngeal electromyography (LEMG), is very poor or that life expectancy is very short (so the patient cannot wait for spontaneous voice recovery). Mau et al. (2017) and Mau (2019) also suggested that the probability and speed of spontaneous recovery are dependent on the severity of the nerve damage as well as the distance of the site of the nerve injury to the vocal muscle. Patients with idiopathic paralysis, mostly due to neurapraxia, would therefore be to expected to recover more frequently and more quickly than those with neurotmesis or axonotmesis following surgery.

A second key question is what to propose as an effective intervention for UVFP. The time course of the injury, the severity of the nerve damage and its location can therefore guide the choice of a treatment option. In addition, the position of the paralyzed vocal fold is a determining factor as it influences the severity of vocal, respiratory and/or swallowing symptoms. Lastly, although some recommendations are reported in this introduction, these do not prevail over the patient’s needs and expectations in determining the most appropriate treatment option. Permanent interventions recommended after a waiting period of 9–12 months will not be discussed here. In the waiting period, two types of interventions could be offered to patients, behavioral voice therapy and injection augmentation. Reviews of the literature supported that behavioral voice therapy is effective in improving the voice of patients with UVFP as well as avoiding the development of maladaptive compensatory vocal behaviors (Walton et al., 2017; Maryn et al., 2020). A majority of studies on the behavioral voice therapy for these patients reported improvement in voice quality or glottic closure (the paralyzed vocal fold moving into a more favorable position for the voice). Only the study of Mattioli et al. (2015), discussed results in terms of improvement of mobility of the paralyzed vocal fold. Since there was no control group and since the improvements of the paralyzed vocal fold were mainly observed in the early behavioral voice therapy groups (before 2 months), authors agreed that it was difficult to distinguish the part of the progress related to the therapy and the part related to possible spontaneous reinnervation. In a more complex way, it is also possible to hypothesize that early intervention might support this process, but this cannot be confirmed. The second intervention option, augmentation injection of a temporary and fully resorbable material, allows to improve the patient’s voice immediately by filling the paralyzed vocal fold (Courey and Naunheim, 2020; Wang C. C. et al., 2020), placing it in a more medial position and thus more favorable position for glottic closure during phonation. This is an effective and temporary procedure. The time of complete resorption, ranging from 1 month to 1 year depends on the injected material (Kwon and Buckmire, 2004). Furthermore, this intervention does not prevent the reinnervation process (Marques et al., 2021). Several retrospective studies, have suggested that early injection would reduce the need for permanent intervention at the end of the waiting period (Mau, 2019). However, most of these studies did not specify whether this decrease was related to a recovery of a satisfactory voice in the absence of vocal fold mobility or to a recovery of vocal fold mobility. Furthermore, except for the prospective study of Pei et al. (2015), no experimental design has attempted to evaluate the possible reinnervation process (Mau, 2019). Pei et al. (2015) showed that 6 months after the injection, there was no difference in quantitative LEMG (peak turn frequency measure) between an injection group and a control group. They also emphasized that the decision to perform a permanent intervention is usually influenced by other considerations besides the voice function, such as the patient’s general and mental health, their confidence in the medical procedure, and the interactions with their physician. Therefore, the permanent intervention rate would not be a reliable indicator of recovery. This study did not detail how many patients had recovered voice and/or mobility in the two groups at the end of 6 months. Besides, in the injection group assessments were also performed at 1 and 3 months, but not in the control group. It would have been interesting if these results had been detailed and compared with similar assessments in the control group. Indeed, it may be considered that an injection of a temporary material could generate modifications or influence the recovery process differently than natural recovery, even if the final state is similar.

Different hypotheses have been suggested as to how early intervention might promote spontaneous recovery of voice and/or mobility. Accordingly, it would be interesting to analyze what happens at the level of the peripheral nervous system, but also at the level of the brainstem and the brain; neuroplasticity required for such hypotheses, occurring more central than the recurrent laryngeal nerve.

Only four studies have used Magnetic Resonance Imaging (MRI) to investigate changes that occur in patients with vocal fold paralysis. The study of Kiyuna et al. (2020) compared a group of 12 patients with left UVFP for more than 6 months (*M* = 21.17 months, SD = 22.6) to a group of 12 matched control subjects sustaining 3 s. [i:] vowel in an MRI scanner. The comparison of these two groups between rest and phonation revealed that the UVFP patients showed increased brain activation in the following regions: in the right secondary motor areas (BA 6), primary somatosensory areas (BA 1 and 2), angular gyrus (BA 39), bilateral SMA (BA 6), left inferior parietal lobule (BA 40), superior parietal lobule (BA 7), and middle frontal gyrus (BA 8). The right superior temporal gyrus (BA 22) showed reduced brain activity in UVFP patients. This shows that, in case of chronic vocal fold paralysis, sustained vowel phonation involves an hyperactivation of the phonatory motor network and that a peripheral nerve damage leads to neuroplasticity. Furthermore, two case studies investigated brain changes following voice improvement. Galgano et al. (2009) investigated central neural activation changes after a permanent intervention (type I medialization thyroplasty) in a patient with an UVFP for 3 months. fMRI scans were completed prior to surgical rehabilitation, 1 month following surgery and 6 months following surgery during following four tasks: a sustained “uh” phonation task at high pitch, sustained “uh” at comfortable pitch, sustained “uh” at low pitch, and a repetition task of the “uh” sound over 4 s. Increased activation was reported in premotor planning (middle frontal gyri) and motor execution areas (precentral gyri) in the frontal lobe, in the inferior and superior parietal lobes, in the superior temporal gyrus, in the thalamus and in the cerebellum 1 month post-surgery. The results at 6 months, however, were difficult to interpret because the patient’s health had deteriorated significantly and she was undergoing chemotherapy. Joshi et al. (2011) selectively blocked (i.e., temporarily paralyzed) the right recurrent laryngeal nerve by injecting a solution of lidocaine and epinephrine in this nerve. Brain activations were compared in a sentence reading task before induced paralysis, during paralysis and 1 h after recovery. Greater activation was reported during recovery phase compared to baseline or paralysis period. Although this was different from pathological nerve damage leading to more chronic UVFP and the recovery process was quick, this study showed evidence that neuroplastic changes were observed directly after paralysis and recovery. Perez et al. (2020) compared a group of 10 patients who presented UVFP for more than 12 months and who had been treated with a permanent intervention (type I medialization thyroplasty) for at least 3 months, to a group of 12 control subjects. They investigated resting-state connectivity and reported significant differences in connectivity between the two groups: increased resting-state connectivity between both caudate nuclei and the precuneus and decreased connectivity between these nuclei and the left cerebellar hemisphere, for UVFP patients. Their study testifies that some long-term changes underlying learning processes can be observed in resting state. These studies indicate that (a) UVFP triggers different phonatory activation patterns than that observed in healthy subjects and that (b) improvement of voice following intervention may lead to adapted phonatory brain function.

Considering this information, and in an attempt to improve understanding of the spontaneous reinnervation process, as well as the possible impact of early interventions on voice recovery and/or vocal fold mobility, it appears interesting to evaluate peripheral and central neuroplasticity at several time points, such as shortly after nerve injury and subsequently for a period up to 9/12 months. Therefore, the present study followed three UVFP patients for 1 year with several multiparametric voice assessments and MRI. First, we will comment on the task proposed in this study with regard to our previous review of the literature on activated regions in sustained vowel phonation tasks (Dedry et al., 2022). Second, we will describe the evolution of brain and brainstem activations for this phonation task in three UVFP patients according to time, proposed interventions and vocal and/or nervous recovery. Third, we will focus on the neural recovery of the two patients who recovered vocal fold mobility. Finally, we will look at how the resting-state connectivity was related to the other results. Due to this small number of patients (as a consequence of hindered recruitment during the COVID-19 pandemic), we were restricted to an exploratory and qualitative multiple-cases study.

## Materials and methods

### Participants

The protocol of this prospective multiple-cases longitudinal study was approved by the Ethics Committee of the University Hospital of Saint-Luc (number: B403201837695) and was conducted in accordance with the principles of the Declaration of Helsinki. All participants signed a written informed consent. The inclusion criterium for the study was to have had a UVFP for less than 3 months.

Three women with UVFP and one healthy control participant with no history of neurologic, hearing or voice disorder were enrolled. The first patient (P1) was a 61-year-old left-handed female with left UVFP with vocal fold in abductory position for 87 days at the time of study inclusion. The identified cause of her paralysis was the excision of a mediastinal paraganglioma. The second patient (P2) was a 30-year-old right-handed female and had a right UVFP in paramedian position resulting from a thermoablation of a thyroid nodule 78 days before inclusion in the study. The third patient (P3) was a 54-year-old and right-handed female with a right UVFP in abductory position. She identified the onset of symptoms as following an upper respiratory tract infection that had occurred 41 days prior to study inclusion. The female control participant (C) was right-handed and 55 years old. All participants were French speaking.

### Intervention and assessment procedures

The three patients received different treatments and had several multiparametric voice assessments during the 9 months of their participation. The healthy control participant received no intervention.

In terms of treatment, P1 had fifteen 30-min sessions of voice therapy as well as video-guided homework to be performed between sessions. The sessions were scheduled over 10 weeks (two sessions per week for 5 weeks and then one session per week for the next 5 weeks). The objective of the therapy was to progressively improve the opening and closing movements of the vocal folds by exerting the intrinsic abductor and adductor muscles of the larynx. This therapy included resonant voice exercises, pitch and loudness variation training, glottal fry exercises, humming exercises, soft glottal closure exercises, phonation on inhalation, Valsalva training, sustaining vowel phonation and sniffing/smelling exercises. At the beginning of vocal therapy, P1 also received a sham injection, which consisted of a subdermic injection of saline solution in the neck (supra-hyoid puncture site), under conditions similar to an acid hyaluronic augmentation injection (described below). Sham interventions (sham injection or sham voice therapy) were set up so that each patient would receive two similar interventions without knowing which one and whether they were effective and specific to UVFP.

P2 received a sham voice therapy consisting of exercises that were designed to not actively mobilize the vocal folds. These exercises aimed at avoiding maladaptive compensatory behaviors, promoting neck and shoulder muscles relaxation and optimizing breathing and posture. The planning of the sessions, their duration and the homework requirements were identical to the protocol of P1. P2 was intended to receive a hyaluronic acid injection but due to anatomical reason (thyroid gland enlargement), the injection could not be performed during the laryngology consultation, hence she also received a sham injection. Therefore, P2 did not receive any effective or specific treatment for UVFP.

P3 underwent vocal fold augmentation with a supra-thyroid injection of 1 ml of hyaluronic acid under local anesthesia and controlled by laryngoscopy in the right/paralyzed vocal fold. She also had the same sham voice therapy as P2.

**FIGURE 1:**
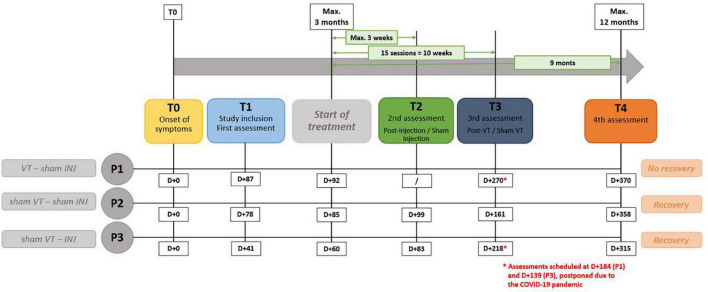
Description of experimental procedure. “VT” means voice speech therapy and “INJ” means injection. D + 0 is the presumed day of the nerve damage. “T” represents the different time points of the study.

In terms of timing, the COVID-19 pandemic has disrupted the originally planned timelines for assessments; P1 had three assessments while P2 and P3 had four. Considering that day + 0 is the presumed day of the nerve damage, P1 had three assessments at D + 87, D + 270, and D + 370. Voice therapy was started at D + 92. P2 had four assessments at D + 78, D + 99, D + 161, and D + 358. Finally, P3 had four assessments at D + 41, D + 83, D + 218, and D + 315. The hyaluronic injection occurred at D + 60. The delays in days from the paralysis to the different follow-up points are represented in Figure 1.

### Unilateral vocal fold paralysis assessment

Multiparametric assessment as recommended by Dejonckere et al. (2001) and Mattei et al. (2018) was carried out at three or four moments.

Videostroboscopic examination confirmed UVFP and allowed to qualitatively track the evolution of the following parameters with visual analogue scale: glottic closure, position of vocal folds, mucosal wave and vibratory amplitude, regularity and symmetry. Participants were asked to breathe, to sustain [a:], to produce [i:] at high pitch, to repeat three times the sentence “Le petit chat fait sa toilette” and to sniff. These examinations were then scored by three experienced otolaryngologists in order to classify the mobility of the vocal fold on the Ricci-Maccarini et al. (2018) 6-point scale (1: immobile in median position, 2: immobile in paramedian position, 3: immobile in intermediate position, 4: immobile in abducted position, 5: hypomobile, 6: normally mobile). In case of disagreement, the score proposed by 2 of 3 raters was retained.

The following acoustic measures were collected: intensity range, mean fundamental frequency, fundamental frequency range, jitter, shimmer, noise-to-harmonic ratio, smoothed cepstral peak prominence. The following aerodynamic measures were collected: maximum phonation time, phonatory quotient, estimated subglottic pressure, mean air flow (MeAF). The collection procedure of these measures is described in the Supplementary Table 1. Multiparametric indices such as the Acoustic Voice Quality Index (v.02.02) (Maryn et al., 2010) and Dysphonia Severity Index (original version) (Wuyts et al., 2000) were calculated. The Hirano’s GRBASI scale (Hirano, 1989) and the Voice Handicap Index-30 (Jacobson et al., 1997) were completed.

Finally, laryngeal electromyography (LEMG) of the right and left thyroarytenoid and cricothyroid muscles was performed using concentric needle electrodes with a recording area of 0.07 mm^2^ (length = 37 mm, diameter = 0.46 mm), connected to the Nicolet Viking AT2 + 6 amplifier electromyography acquisition system (Natus Medical Incorporated, Pleasanton, CA, United States). For thyroarytenoid muscles, recordings were made at rest, during sustained phonation of the [a:] at habitual pitch and comfortable loudness for at least 3 s and during a series of three sniff inspirations. Potentials in the cricothyroid muscles were measured during phonation of a high-pitched [i:]. Qualitative LEMG interpretation was completed by three professionals (one neurologist, one otorhinolaryngologist and one speech-therapist), based on the characteristics of the EMG waveforms described in Kneisz et al. (2020) (high-pass filter set to 20 Hz, low-pass filter setting 10 kHz, sampling frequency set to 20 kHz). Only the recording of the thyroarytenoid muscle on the paralyzed side during the sustained phonation task was qualitatively scale-coded. The volitional electromyographic activity during phonation in the thyroarytenoid muscle of the paralyzed side was scored on a 4-point scale (1: dense volitional activity, 2: midly decreased volitional activity, 3: strongly decreased volitional activity, 4: single fiber activity) (Kneisz et al., 2020). Again, in case of disagreement, the score proposed by 2/3 was retained.

During the first assessment, a hearing test was also performed (tonal and vocal audiometry). At this time, if patients had a hearing impairment, were unable to produce the sustained [a:] for 3 s or did not have an electromyographic signal in the thyroarytenoid muscle during the LEMG, they would have been excluded from the study. For evident reasons, patients with contraindications for MRI examinations could not be recruited. This was never the case in this study.

### Neuroimaging assessment

#### Functional magnetic resonance imaging tasks

Before each fMRI scanning session, participants had a 15-min appointment with the experimenter to record the sustained [a:], to review the task and to practice it outside the MRI scanner. Participants were asked to sustained [a:] on habitual pitch and intensity levels for 4 s. This sustained phonation was recorded and was then edited to retain 3 s of continuous sound (the onset of the production was removed from the recording). The sustained [a:] vowel was chosen because it is generally used in the laryngology/voice clinics for acoustic and aerodynamic measurements as well as during videostroboscopy and LEMG. The short 3-s duration was chosen to ensure that all patients would be able to produce the sustained [a:] at all times during the time course of their vocal fold paralysis.

The experimental protocol consisted of three conditions. For the PHONATION_AUDITION condition, participants were asked to produce the [a:] on habitual pitch and intensity for 3 s. To ensure sufficient auditory feedback of the sound (in addition to bone conduction) in the noisy MRI scanner, the prerecorded sustained [a:] was also played through MRI compatible audio headphones simultaneous with the voice production. Participants were told that, in this condition, they were hearing herself live. In the AUDITION condition the participants were asked only to listen to their pre-recorded sustained [a:]. In this condition, they had no phonatory task. In the REST condition, the participants had nor phonatory nor listening task.

#### Functional magnetic resonance imaging paradigm and procedure

The fMRI experiment was presented with a randomized event-related design. Each condition was repeated 15 times for PHONATION_AUDITION and AUDITION conditions and 10 times for REST condition. The run total duration was 11 min 3 s.

**FIGURE 2:**
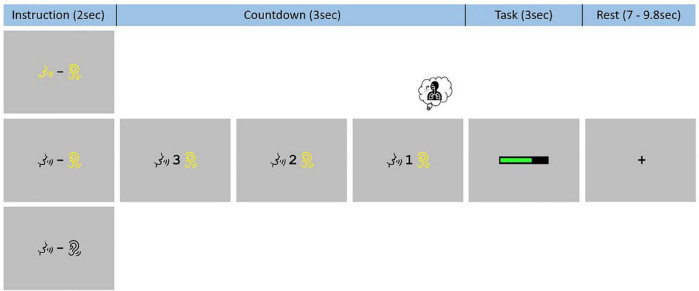
Functional magnetic resonance imaging task paradigm. Participants were trained to systematically inhale on the “1” of the 3-to-1 countdown and had to keep the mouth slightly open, in the articulatory position for the [a:] phonation, during the whole experiment.

Each event began with the presentation of an instruction slide for 2 s. On this slide, the same two visual symbols were presented to participants in all three conditions: a talking mouth and a hearing ear. When these symbols were presented in black color (on a gray background), no “phonation” and “audition” should be performed (REST). When they both were in yellow color, participants had to listen to the sustained [a:] while producing it (PHONATION_AUDITION). When only the hearing symbol was in yellow color, participants only need to listen to their prerecorded sustained [a:] (AUDITION). After the instruction slide, there was a visual countdown from 3-to-1 before presentation of a green loading bar that progressed during the 3 s of the task. Finally, there was a rest period ranging between 7 s and 9.8 s with a black fixation cross. An example of such sequence is demonstrate in Figure 2. These visual instructions were generated using Eprime 2.0.8.90 (Psychology Software Tools, Pittsburgh, PA, United States^1^) and presented on a screen (NordicNeuroLab, Norway^2^). A MRI-compatible headset was needed to attenuate the background noise and play the pre-recorded sustained [a:] (NordicNeuroLab, Norway, see text footnote 2). A coil angle-mirror was used to allow participants to see the screen located over the head of the subject.

Two specific instructions were given to reduce non-experimental variability between all three conditions due to articulatory and respiratory movements. Participants were instructed to place themselves in the articulatory position for the [a:] phonation at the beginning of the task and to remain with their mouths slightly open during the entire functional acquisition. They were also asked to systematically inhale on the “1” of the 3-to-1 countdown regardless of the condition.

In the absence of an MRI-compatible microphone, it was not possible to monitor the correct execution of the task during or after the examination. Different specific questions about the procedure were therefore asked to the participant directly after the examination to confirm the correct completion of the tasks in the three conditions.

#### Imaging acquisition parameters

Anatomical, functional (resting-state and task) and multishell diffusion sequences were acquired at the Cliniques Universitaires Saint-Luc (UCLouvain, Belgium) using a 3T head scanner (Signa Premier, General Electric Company, United States) equipped with a 48-channel coil. Diffusion images were not used for the current study. A three-dimensional (3D) T1-weighted data set encompassing the whole brain was selected to provide detailed anatomy (1 mm^3^) thanks to a MPRAGE sequence (inversion time = 900 ms, repetition time (TR) = 2188.16 ms, echo time (TE) = 2.96 ms, flip angle (FA) = 8°, field of view (FOV) = 256*256 mm^2^, matrix size = 256*256, 156 slices, slice thickness = 1 mm, no gap, total scan time = 5 min 36 s). Task and resting-state MRI T2-weighted sequences of brain activity were collected with echo-planar imaging: FOV = 220*220 mm^2^, matrix size = 110*110, TE = 30 ms, TR = 1700 ms, FA = 90°, 75 slices (order ascending and interleaved), slice thickness = 2 mm, parallel imaging (ARC2) and hyperband factor = 3. For the task sequence, the whole brain slices were scanned 390 times per run (= 11 min 3 s) and for the resting-state, the whole brain slices were scanned 210 times per run (= 5 min 57 s).

### Neuroimaging data processing

The MRI data were analyzed using BrainVoyager (Version 22.2.2, Brain Innovation, Maastricht, Netherlands). Preprocessing of the resting-state and functional data consisted of linear trend removal to exclude scanner-related signal drift, a temporal high-pass filter to remove frequencies lower than 0.07 Hz (task) and 0.005 Hz (resting-state) and correction for head movements using a rigid body algorithm for rotating and translating each functional volume in 3D space. The data were also corrected for time differences in the acquisition of the different slices. For the resting-state, because spontaneous low-frequency fluctuations are not exclusively BOLD-related fluctuations, but also contaminated by non-neural signals (i.e., artifacts), several additional pre-processing steps were added to remove these undesirable sources of variance. Regression analyses were performed to remove artifacts due to residual motion (the six movement regressors were obtained during the previous motion correction) and changes in ventricles (the signal from the ventricular mask defined in each participant). The data were smoothed in the spatial domain (Gaussian filter, FWHM – 5 mm). To compare the locations of activated brain areas across participants, all anatomical and functional volumes were spatially normalized in Montreal Neurological Institute – MNI space and flipped for P1, and the statistical maps computed were overlaid on the 3D T1-weighted scans. In this way, the acquisitions of the three patients are consistent with paralysis of the right vocal fold. All co-registrations were verified and movement corrections were optimized, using a sinc interpolation.

### Data analyses

Since all MRI scans acquired for P1 were flipped (inverted), all analyses assumed right UVFP. Furthermore, the analyses were performed for a particular contrast, the PHONATION contrast, which includes the activations of the PHONATION_AUDITION condition fMRI scans minus the activations of the AUDITION condition scans (PHONATION = [PHONATION_AUDITION – AUDITION]). The MOBILE map was also investigated independently of the PARALYZED map. The MOBILE map was computed by grouping the “mobile” time points; these were sessions 3 and 4 of P2 and P3, as well as the four sessions of C1 (8 runs). The PARALYZED map included the three sessions of P1, as well as session 1 and 2 of P2 and P3 (7 runs). Besides, four behavioral measures were also selected for analysis of correlations with MRI observations. These were selected because they represented different aspects of the voice and vocal fold mobility. The Acoustic Voice Quality Index – AVQI (Maryn et al., 2010) is an acoustic measure, the mean air flow (MeAF) is an aerodynamic measure, the scale-coding of qualitative LEMG is a peripheral nerve measure and the qualitative laryngoscopy scale is a visual measure of vocal fold mobility. These are provided in Table 1 for the different time points of the study.

**TABLE 1:** Behavioral measures at different assessment times.

	**AVQI**	**MeAF**	**LEMG**	**Laryngoscopy**
P1_T1	6.75	0.23	4	4
P1_T3	7.03	0.26	3	3
P1_T4	5.83	0.32	3	3
P2_T1	4.63	0.21	3	2
P2_T2	4.62	0.16	3	2
P2_T3	4.70	0.22	4	5
P2_T4	5.51	0.19	1	5
P3_T1	6.73	0.61	4	2
P3_T2	4.54	0.23	4	2
P3_T3	4.39	0.16	1	6
P3_T4	6.28	0.10	1	6

The Acoustic Voice Quality Index – AVQI (Maryn et al., 2010) is a multiparametric acoustic indicator rated between 1 and 10. The higher it is, the lower the voice quality. The Men Air Flow (MeAF) is an indicator of the airflow used for the production of a voiced sentence (in L/sec). For laryngeal electromyography, the volitional activity in the thyroarytenoid muscle of the paralyzed side was scored on a 4-point scale (1: dense volitional activity, 2: midly decreased volitional activity, 3: strongly decreased volitional activity, 4: single fiber activity) (Kneisz et al., 2020). For laryngoscopy, the mobility of the paralyzed vocal fold was assessed based on Ricci-Maccarini et al. (2018) 6-point scale (1: immobile in median position, 2: immobile in paramedian position, 3: immobile in intermediate position, 4: immobile in abducted position, 5: hypomobile, 6: normally mobile).

#### Functional magnetic resonance imaging analysis

Functional data were analyzed using a multiple regression model (general linear model; GLM) consisting of predictors, which corresponded to the particular experimental conditions: PHONATION_AUDITION, AUDITION and REST. The predictor time courses used were obtained by convolution of a condition box-car time course with a standard hemodynamic response function (two-gamma HRF). We conducted two types of analyses, based on regions of interest (ROI) and in the whole brain.

To define the regions of interest (ROI) (Dedry et al., 2022), the present experiment used a previous literature review. This study highlighted 20 left-sided coordinates or clusters of activation and 23 right-sided coordinates or clusters of activation in fifteen different regions in the brain and cerebellum (named according to the atlas of Mai et al., 2006). In this previous literature review, it was decided to exclude regions that were cited only once in the articles included in the literature review from the qualitative analysis. These 28 regions were added to our analyses to ensure a complete overview. Finally, we chose to include two coordinates in the brainstem since no study had investigated it so far. All ROI were created by generating a spherical volume of interest around these coordinates with a radius of 5 mm (515 mm^3^). The region of the brainstem identified was the nucleus ambiguus since it is the first motor relay of the recurrent laryngeal nerve. MNI coordinates of this nucleus have been determined from the obex thanks to the atlas of Paxinos et al. (2012) and were validated by a neurosurgeon. The solitary tract nucleus, as first sensory relay of the superior laryngeal nerve and the recurrent laryngeal nerve, was also examined. However, since MNI coordinates of the nucleus ambiguus (from ±5, –40, –49 to ±5, –40, –67) were so close to those of the solitary tract nucleus (±2, –46, –58) (Frangos and Komisaruk, 2017) and as the radius of the volume of interest was 5 mm, it was decided to consider this location as a single region: the motor/sensory nuclei of voice, located in the medulla oblongata of the brainstem. A total of 73 ROI were used for the analyses (cfr. Supplementary Table 2). First, to validate the sustained vowel phonation task performed in the MRI scanner with regard to the selected ROI, analyses were conducted to highlight significant brain activation for the PHONATION contrast in the 73 ROIs for the MOBILE map (one-sample Student’s *t*-Test). Second, for P2 and P3 separate analyses were run to identify the ROI in which the MOBILE times (3rd and 4th sessions) and the PARALYZED times (1st and 2nd sessions) ([MOBILE – PARALYZED]) showed significant differences in activation (independent sample Student’s *t*-Test). To further investigate the MOBILE versus PARALYZED differences, correlational analyses (Pearson correlation) between behavioral measures and beta-weights in the ROIs were also run for P2 and P3.

In order to complement, a whole brain analysis was conducted for each participant and for each assessment time point, to determine, without *a priori* functional localization, the involved regions for the PHONATION contrast. The fifteen maps (3 × P1, 4 × P2, 4 × P3, 4 × C1) were corrected for multiple comparisons using Bonferroni correction (*p* < 0.05).

#### Resting-state functional connectivity analyses

We used BrainVoyager and a customized Matlab code (The Mathworks) to calculate cross-correlations between the average time-course signals, extracted from 55 ROI (cfr. Supplementary Table2). Fifty-four regions were derived from the 73 ROI (described in the previous paragraph) intersected with the MOBILE map. The region of voice motor/sensory nuclei in the brainstem was included in these 54 ROI on the right but not on the left; we therefore chose to add the left nuclei region. This resulted in 1,485 pairs of resting-state functional connectivity per subject. We entered these pairs in an ANOVA to investigate differences between the MOBILE and PARALYZED maps. Independent sample Student’s *t*-Test was also conducted between the MOBILE and PARALYZED times particularly for P2 and P3. For P3, correlational analyses using Pearson’s *r* coefficient were conducted between behavioral measures and ROI.

## Results

### Validation of the sustained phonation task

**FIGURE 3:**
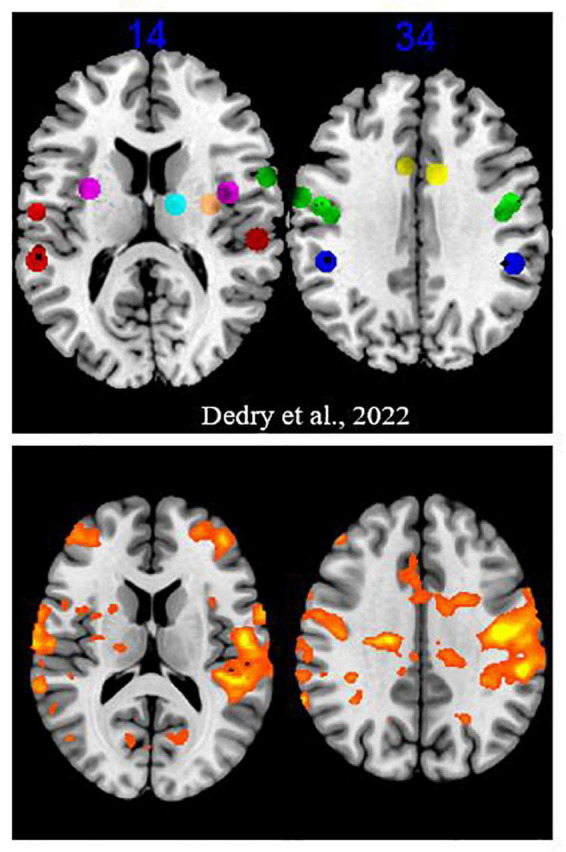
Activation map for the “Mobile” runs. The upper part is excerpted from a figure in the Dedry et al. (2022) review of literature. The clusters and coordinates of interest were represented on a brain map at two Z coordinates in MNI. The colors were assigned as follows: green = frontal lobe, red = temporal lobe, dark blue = parietal lobe, yellow = cingulate gyrus, purple = insula, orange = putamen, light blue = thalamus. The bottom part reports activation at a liberal significance threshold (*p* = 0.05) for the “MOBILE” runs of the present experiment.

Regions activated for the PHONATION contrast when the MOBILE runs were pooled, compared to forty-three coordinates or clusters of activation highlighted in our literature review of fMRI sustained vowel phonation tasks (Dedry et al., 2022), are illustrated in Figure 3. When examined, 35/43 ROI were significantly activated or contained an activation peak (*p* < 0.05). Non-activated clusters were the following: OP47_InfFrontF_orbit_L1, OP47_InfFrontF_orbit_R3, BA42_PlanTemp_Cl_R1, Insula_L1, Putamen_L1, Thalamus_L1, Thalamus_R2, and Cerebellum_CrusII_R4. With the exception of the cerebellar Crus II, other ROI in the inferior frontal gyrus, planum temporale, insula, putamen and thalamus showed activation. Furthermore, when considering the PARALYZED map, the cluster in the left putamen was activated. This does not imply that these regions could not be activated individually for a participant.

To further validate the ROI of the present study, the thirty newly added ROI were also examined. Six did not present any significant activation (*p* < 0.05) (BA8_SupFrontG_Cl_L1, BA8_SupFrontG_R1, BA37_MidTempG_L1, BA37_MidTempG_R2, PiriformCortex_R1, AmygdaloidIsland_L1). Two other ROI in the middle temporal gyrus (BA37, BA21) showed activation. Brodmann area 8 in the superior frontal gyrus and the amygdalian areas did not appear to play a role in phonation.

### Brain activation profiles over time

**FIGURE 4:**
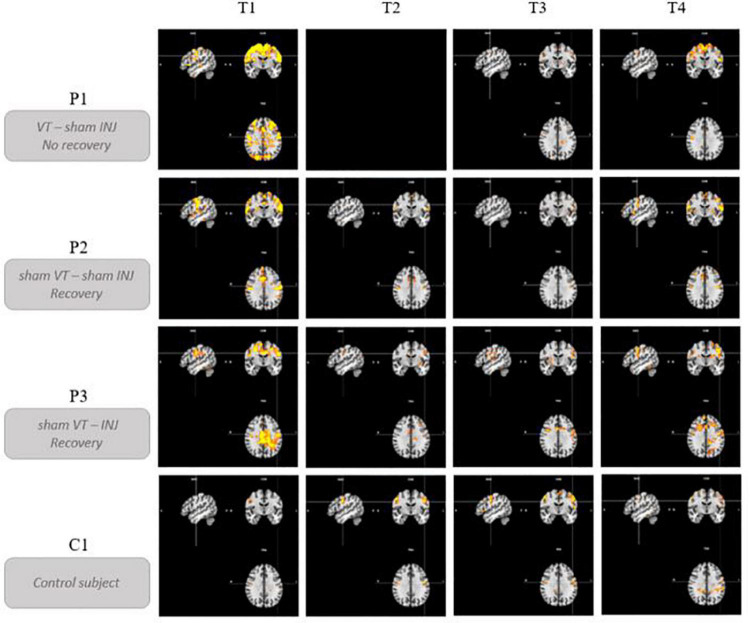
Brain activation profiles over time. Evolution of brain activations for the PHONATION contrast using a Bonferroni correction for each patient and the control subject over time. “VT” means voice speech therapy and “INJ” means injection. “T” represents the different time points of the study. These images are centered on the left laryngeal motor cortex cluster located at [–50, –8, 32] (in MNI coordinates).

Figure 4 illustrates the evolution of brain activations for the PHONATION contrast using a Bonferroni correction for each patient and the control participant over time. Only the second MRI scan was missing for P1. These images are centered on the only cluster output from the ALE meta-analysis performed in the previous literature review (Dedry et al., 2022), located at [–50, –8, 32] (in MNI coordinates). This coordinate corresponds to the dorsal laryngeal motor control area and more precisely, the ventromedial peak (Brown et al., 2008, 2009; Belyk et al., 2021).

Qualitatively, hyperactivation was observed for the three patients at the first fMRI session (between 41 and 87 days post-paralysis) in comparison with the control participant. This hyperactivation was mainly localized bilaterally in the premotor, motor and somatosensory integration regions of the frontal lobe (pre- and post-central gyri – BA1, BA2, BA4, BA6, BA43), in the cingulate gyrus (BA24, BA32) and also but less consistently, in the parietal lobe (supramarginal gyrus and parietal operculum – BA40). In general, there was a decrease in activation at times 2 and 3. Time 4, which corresponded to stable state (reinnervation process no longer occurring because the delay was about 1 year after the nerve injury), resulted in more activation than at times 2 and 3 but not in the hyperactivation observed at time 1.

P1, who did not recover vocal fold mobility, is the patient showing the most activation on the last session, mainly in the same frontal regions as at time 1. Unlike P2 and P3, she already had activations in the superior frontal gyrus at time 1 that were still present at the final time. P2 and P3 recovered vocal fold mobility between the second and the third session. Different recovery profiles can be considered. P2, who recovered mobility without injection between time 2 and time 3, showed a progressive bilateral reactivation (on the third and the fourth fMRI scans). Her last scan was more similar to that of the control participant. P3 recovered mobility (between time 2 and time 3) following an injection of hyaluronic acid just before time 2 in the right vocal fold. Her recovery profile seems to be lateralized with a more important activation in the left hemisphere.

**FIGURE 5:**
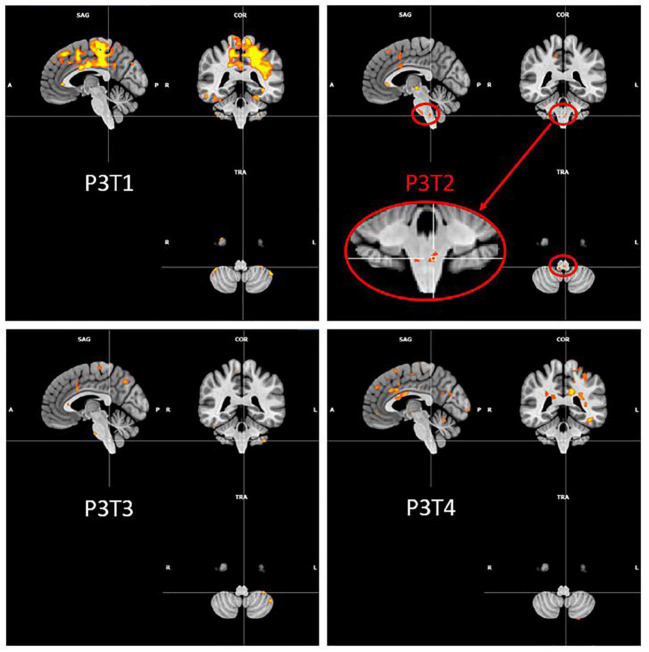
P3 over time activation of the region of ambiguus and solitary tract nuclei. Evolution of Patient 3 activation in the region of ambiguus and solitary tract nuclei for the PHONATION contrast using a Bonferroni correction. These images are centered at [–5, –40, –52] (in MNI coordinates).

It is also interesting to look at what happens in the region of the nucleus ambiguus (that is the first central motor nervous relay) and of the solitary tract nucleus (that is the first central sensory nervous relay) of the vagus nerve (cfr. Figure 5). No activation was detected in this region, for any participant and at any time, except for the second fMRI scan for P3, that was acquired 23 days after the injection of hyaluronic acid in the right vocal fold. Although the injection was unilateral, the activation observed in these nuclei was bilateral and predominantly on the left side. This activation did not last in time, since it was no longer present at times 3 and 4.

### Brain activation mobility recovery profiles for P2 and P3

Given these differences, the recovery profiles of P2 and P3 were investigated in more detail by highlighting the activation differences in the 73 ROI between the MOBILE runs (first and second MRI sessions) and the PARALYZED runs (third and fourth MRI sessions) for the PHONATION contrast. Independent samples Student’s *t*-Test was run and only significant differences (*p* < 0.05) are reported below. The reason for grouping the times two by two was to increase the statistical power. For a detail of the individual times, please refer to Figure 4. To support the activation variations observed in some of these ROI, correlations with behavioral measures were investigated. Only Pearson’s *r* coefficients greater than *r* = 0.6 were reported, none of them were significant since with four assessment times, the correlation should have been *r* = 0.95 to reach significance (*p* < 0.05).

For P2, three ROI deactivated significantly when reinnervation had occurred and the vocal fold was mobile. These were the following ROI: BA6_PreG_Cl_R2, BA37_MidTempG_R2, PostCingulateG_R3. No correlation was observed with MeAF, AVQI or the scale-coding of qualitative LEMG. Deactivation of these three regions correlated negatively with the qualitative laryngoscopy scale.

P3 had a more complex profile. She presented deactivations but also significant activations when the vocal fold was mobile. First, deactivations were observed in the following ROI: BA4_PreG_R2, BA4_PreG_L3, BA4_PreG_Cl_R3, BA6_ParacentralLobule_R2, BA7_SupramarginalG_R1, Thalamus_R1, Brainstem_MotSens_L. However, this deactivation observed in the voice motor/sensory nuclei in the brainstem was probably only due to the isolated post-injection hyperactivation at time 2. These deactivations correlated positively with (a) the MeAF (*r* from 0.73 to 0.88) in the precentral gyrus and paracentral lobule (4/7 ROI), (b) AVQI (*r* from 0.69 to 0.87) in 3 of these 4 deactivated regions (except BA4_PreG_Cl_R3), and (c) negatively (*r* = –0.61) with the deactivation of the voice motor/sensory nuclei in the brainstem. As explained above, since these nuclei were never activated except after injection, this correlation must be considered with caution. (d) A positive correlation (*r* from 0.61 to 0.66) was also observed with the scale-coding of qualitative LEMG (and reversely with qualitative laryngoscopy scale) in the right precentral and paracentral gyrus, supramarginal gyrus and thalamus (5/7 ROI). Second, when the vocal fold was mobile the following ROI were more activated: BA44_InfFrontG_oper_Cl_R1, OP47_InfFrontF_orbit_L2, BA22_SupTempG_Cl_L1, Insula_L2, Insula_R3, Putamen_R3, Thalamus_R2. A negative correlation (*r* from –0.72 to –0.80) with the MeAF (a) was found for all the regions except the left inferior frontal gyrus. (b) AVQI generally did not correlate with increased activation except for left superior temporal gyrus (*r* = –0.85). (c) The scale-coding of qualitative LEMG correlated negatively and the qualitative laryngoscopy scale positively (r from ±0.67 to ±0.79) with 4/7 ROIs (all except those in the inferior frontal gyrus and the one in the right thalamus).

These ROIs did not contain significant deactivations or activations in the control participant when comparing runs 1 and 2 to runs 3 and 4 except for the BA7_SupramarginalG_R1 that showed a significant difference but in the opposite direction to the one observed in P3 and with a less significant activation peak in the investigated region.

### Resting-state connectivity analyses

#### Differences in connectivity between the MOBILE and PARALYZED maps

**FIGURE 6:**
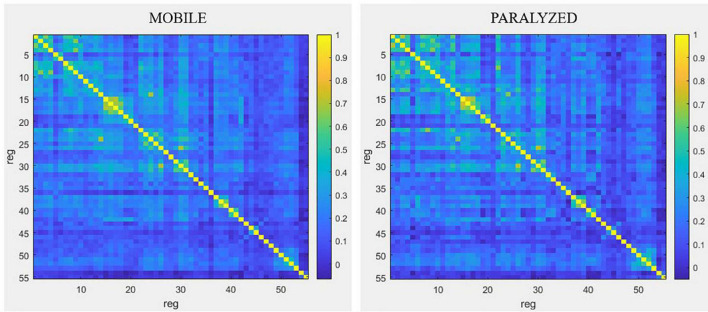
Resting-state connectivity matrices for the MOBILE map and the PARALYZED map. This figure is a symmetric matrix representing the connectivity between the 55 ROI. “reg” means regions. The color scale represents the correlation coefficients from –1 to 1.

Qualitatively, Figure 6 shows that functional connectivity between the 55 ROI selected for resting-state analyses was higher in the PARALYZED map than in the MOBILE map, there were thus more regions whose functional activations vary simultaneously. Quantitatively, only significant (*p* < 0.05) differences will be reported in the text below.

Resting-state connectivity decreased mainly between motor (BA4) and premotor (BA6, BA43) regions and between those regions and the cingulate gyrus and the lobule VI of the cerebellum. Resting-state connectivity also decreased between the parietal regions (BA40, BA7), and between those regions and the motor and premotor regions, insula, putamen and cerebellum. A reduction was also observed between the ROI of cerebellar lobule VI. At the level of the right voice motor/sensory nuclei in the brainstem, resting-state connectivity decreased with the right premotor regions (BA6 and BA43), the right cingulate cortex (BA32), and right lobule VI of the cerebellum. For the left voice motor/sensory nuclei, decreased resting-state connectivity was observed with the left premotor regions (BA43), the right globus pallidus and thalamus, as well as the VI cerebellum lobule. Finally, resting-state connectivity decreased between the insula, putamen, thalamus and cerebellum.

Although decreased connectivity was most often present, some regions showed significant increased functional resting-state connectivity when the paralyzed vocal fold was mobile. The temporal ROI showed mainly increased bilateral resting-state connectivity with each other but also with some right motor (BA4) and premotor (BA6) regions, with the putamen as well as with the post cingulate gyrus.

The highest degree of resting-state connectivity changes (either increase or decrease) between MOBILE and PARALYZED maps was found in the following six ROI: Cerebellum_VI_R1, BA43_PreG_Cl_L1, BA7_SupramarginalG_R1, Cerebellum_Cl_VI_L1, BA42_PlanTemp_R2, and Brainstem_ MotSens_L.

#### Differences in connectivity at the brainstem level for P3

Following the observation of activation for P3 just after injection in the area of the voice motor and sensory nuclei in the brainstem, it appeared interesting to investigate the modification of functional resting-state connectivity between these nuclei (left and right) and other ROI. MeAF showed the highest rate of correlation with changes in resting-state connectivity between the brainstem nuclei of interest and other regions. Moreover, the variations of the MeAF were important for P3 and reflected the immediate effect of the received injection. Having an UVFP in abduction, the MeAF of P3 was very high at time 1 (0.61 L/s) due to glottal air leakage. Immediately after the injection, at time 2, an important decrease of the MeAF was observed (0.23 L/s) due to mechanically restored glottic closure (by filling the paralyzed vocal fold with hyaluronic acid). At times 3 and 4, the MeAF continued to decrease slightly (0.16 and 0.10 L/s) and even normalize (Joshi, 2020) due to recovery of vocal fold mobility as confirmed by videostroboscopy. Therefore, we chose to report these correlations. The significant correlations had to reach a Pearson’s *r* coefficient ≥ 0.95. Ten correlations reached this score for the right nuclei and five for the left nuclei. Considering the high number of correlations between *r* = 0.9 and *r* = 0.95 (six on the right and five on the left), we chose to consider them as well.

**FIGURE 7:**
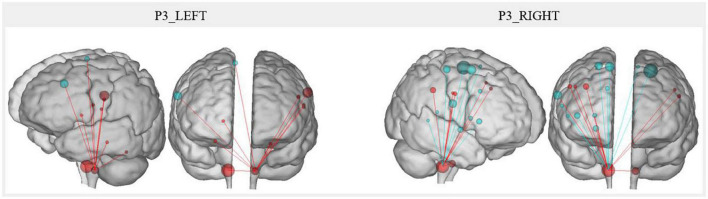
P3 resting-state connectivity changes correlated with the MeAF from the voice motor/sensitive brainstem nuclei. The correlation between the MeAF (L/s) and Patient 3 connectivity changes from the left and right voice motor/sensitive brainstem nuclei is presented. Positive correlations are shown in red and negative correlations in blue. The size of the spheres represents the degree of change in connectivity of each region (either in the direction of more or less connectivity).

The resting-state connectivity changes correlated with the MeAF between voice motor/sensory brainstem nuclei and other regions are illustrated in Figure 7. Qualitatively, right nuclei showed more MeAF correlations with connectivity changes than left nuclei. Also, the left nuclei showed only positive correlations while those on the right had both positive and negative correlations depending on the regions.

For the left nuclei, changes in resting-state connectivity with several regions in the precentral gyrus were positively correlated with MeAF. These ROI were the first cluster in the left laryngeal motor cortex, which corresponded to the ventromedial peak of the dorsal laryngeal motor control area (Brown et al., 2008, 2009; Belyk et al., 2021), and some premotor regions (BA6 and BA43). Connectivity between the left nuclei and the right superior frontal gyrus (BA6) was also positively correlated with MeAF. Similar correlations were also observed with the thalamus and cerebellum. Hence, the correlations were always positive, indicating that the higher the MeAF was (thus, the larger the glottic air leakage), the higher the resting-state connectivity was between these regions and the left nuclei.

For the right side, the resting-state connectivity of the voice motor/sensory nuclei and the two clusters of the laryngeal motor cortex (BA4) correlated with changes in the MeAF. For cluster 1 on the left and bilaterally for cluster 2, which corresponded to the dorsolateral peak of this same region (Brown et al., 2008, 2009; Belyk et al., 2021), connectivity was positively correlated. For the third cluster in BA4 bilaterally, the correlation was reversed. Thus, connectivity was higher when MeAF was low (and glottic air leakage was reduced). This difference in the direction of correlations was also found elsewhere. Resting-state connectivity with the premotor area of the paracentral lobule, the inferior frontal gyrus (BA44), the auditory regions (planum temporale and middle temporal gyrus) and the cingulate gyrus correlated negatively with the MeAF. Consequently, resting-state connectivity was higher when MeAF was low. Conversely, resting-state connectivity with the premotor regions of the precentral gyrus (BA6), supramarginal gyrus, putamen and globus pallidus was increased when MeAF was high. For regions outside the laryngeal motor cortex, laterality was not always assessed because the ROIs did not systematically include identical clusters on both sides.

Finally, changes in resting-state connectivity between right and left voice motor/sensory brainstem nuclei were positively correlated with MeAF. The higher the MeAF was, the higher the connectivity was between the two regions of nuclei.

P2 had an UVFP in paramedian position and her MeAF was within the normal range (Joshi, 2020) from time 1 and relatively stable at all other times (0.21, 0.16, 0.22, and 0.19 L/s). It was therefore clinically irrelevant to analyze the correlations with this measure.

## Discussion

In order to describe the central nervous changes observed in our three patients, part of the analyses of this study were performed using the regions of interest of our previous literature review on sustained phonation tasks in healthy subjects (Dedry et al., 2022). As detailed in the “Results” section, most of the expected regions were also active in the “MOBILE” map of this study. These regions were therefore considered relevant and were used in the analyses.

The qualitative analysis of the different evolutions changes in the three patients revealed a brain hyperactivation for the PHONATION contrast compared to the control participant. This enhanced brain activation was already described in the study by Kiyuna et al. (2020). The authors reported that activity was greater in the regions involved in voice control in patients with chronic UVFP (*n* = 12) compared to the control group. In contrast, Galgano et al. (2009) did not report such significantly increased activation in their patient scanned preoperatively 3 months after onset of UVFP. Our case series study provides an additional precision; hyperactivation seems to take place rapidly after the nerve damage (within 3 months) and seems to be even more increased in this acute phase. Indeed, the activation was very strong at time 1 compared to the other study times. P1, who did not recover vocal fold mobility, was also the one who showed greater activity at time 4. This greater activation compared to the control participant was also observed to a lesser extent for P2 and P3. It would have been interesting to scan these two patients a fifth time to see if, once recovery was stabilized, they returned to a level of activation completely similar to that of the control participant. After 1 year, we cannot state that the recovery of mobility after paralysis activates the brain regions similarly to the activations observed in someone who has never had an UVFP. The resting-state connectivity analyses also provided supplementary and novel information. Indeed, in our patients, the UVFP-related hyperactivation during paralysis observed for the PHONATION contrast in fMRI was associated with an increase in resting-state connectivity (without doing any task) between most regions of interest. Paralysis would therefore lead to an increase in activation in the phonation task but also to an increase in resting-state connectivity between the hyperactivated regions. These resting-state results cannot be compared with those of the only previous study that conducted connectivity analyses (Perez et al., 2020). Indeed, these authors recruited patients who had experienced UVFP for more than 1 year and who had received a permanent intervention. They were outside the time course of nerve recovery. The central plasticity processes at work in their study were therefore quite different from those investigated in the current study.

These qualitative observations also enabled to observe a different brain plasticity in the process of nervous recovery for P2 than for P3. We therefore investigated the changes in ROI activation for the MOBILE time compared to the PARALYZED time. In order to support the assumption that the observed changes were indeed related to the recovery process, we looked at whether these correlated with certain behavioral measures. Few significant changes were observed for P2. These differences were observed on the right side, the “healthy” cerebral side, contralateral to the lesion [since cortico-motoneuronal projections are bilateral but with a contralateral dominance (Jürgens, 2002)]. These were in the direction of deactivation and correlated negatively with qualitative laryngoscopy scale (no correlation with MeAF, AVQI or qualitative LEMG scale-coding). P2, who recovered spontaneously from paralysis in the paramedian position, seemed to show milder and progressive changes. The correlations with behavioral measures were also fewer, but in view of the position in which the paralyzed vocal fold was positioned, the MeAF and the AVQI were already less impacted at baseline. The changes observed in P3 were more frequent, more bilateral, were in the direction of deactivation or activation and were correlated with behavioral measures. Finally, at the final MRI session, activations observed qualitatively for the PHONATION contrast in P2 were more similar to those of the control subject. The profile of P3 showed more activation and a more lateralized activation on the left brain side. A precise analysis or any conclusion with these two patients is not possible, but we hypothesize that the abrupt change induced by the injection may have resulted in greater plasticity and that the post-injection nerve recovery process may differ from the spontaneous nerve recovery process.

It is still a matter of debate whether and how the injection augmentation could promote voice recovery and/or recovery of vocal fold mobility (Mau, 2019). This study is the first to investigate the changes in brain activation following an injection with several MRI scans. We found that, following the injection, the region of the voice motor and sensory nuclei in the medulla oblongata (brainstem) was activated bilaterally. This was the only scan in the study where activation at this location was observed. Furthermore, it is important to note that, at this time, P3 had not yet recovered mobility of the vocal fold (UVFP confirmed at time 2 by videostroboscopy) and that the scale-coding of her thyroarytenoid muscle electromyography was equivalent to time 1. Therefore, there was no indication yet that reinnervation was going to be successful. As this was a multiple-cases study, we applied a Bonferroni correction in order to obtain robuster results. There could also be activation of these nuclei in all mobile vocal fold phonation tasks at a threshold that cannot be reliably assessed with the methodology of this study. It is therefore still uncertain whether this is a one-time activation or a hyperactivation compared to all other assessment times and subjects.

Different hypotheses were summarized by Mau (2019) on the possible impact of injection on voice recovery or vocal fold mobility recovery. Only one of them can be applied to mobility recovery; the injection would restore proprioceptive feedback that would favor spontaneous reinnervation process. The sensory receptors of the muscles of the glottic level would recover sensations similar to those of a healthy voice due to the restoration of the subglottic pressure during phonation and/or due to the vibro-tactile stimulation due to the contact between the paralyzed vocal fold with the healthy vocal fold. Furthermore, auditory perception of clearer and/or louder sounding voice is restored as a result of this early injection (Smith et al., 2020). Restoring feedbacks could therefore play a role in the peripheral reinnervation process, but it remains unclear how these processes occur. Two aspects of our study are in line with the first hypothesis.

First, the activation observed in the nuclei of interest was bilateral but more prominent on the left side, contralateral to the paralyzed vocal fold. The motor and sensory innervation between the vocal folds and the voice-related nuclei in the brainstem being unilateral, it may be surprising to see a bilateral medulla response after an injection into a unilaterally paralyzed vocal fold. One possible explanation is that by injecting the paralyzed vocal fold and restoring the tightness of the glottic closure, the sensation of subglottic pressure and of vocal fold contact would be restored for both vocal folds. Furthermore, the greatest response on the healthy side would be expected since more sensory nerve fibers would be preserved on this side. LEMG at times 1 and 2 confirmed that the nerve damage was low in the vagus nerve pathway and that the superior laryngeal nerve was preserved. Indeed, the signal observed in the crico-thyroid muscle was normal. The middle division of this nerve internal branch is responsible for sensory innervation of the vocal folds (Foote and Thibeault, 2021). Moreover, according to the synthesis written by Foote and Thibeault (2021) on the sensory innervation of the larynx, Galen’s anastomosis and/or the arytenoid plexus are highly prevalent in humans and consequently, the posterior branch of the recurrent laryngeal nerve (classically described as only motor) would play an important role in the sensory innervation of the subglottic region. That this nerve was injured on the paralyzed side and preserved on the mobile side could contribute to a greater sensory response in the left nuclei of the solitary tract.

Second, for Patient 3, MeAF was the measure that showed the highest rate of correlations with changes in resting-state connectivity between the voice-related nuclei in the brainstem and other regions. MeAF is an indicator of the glottic air leakage present during phonation; it is therefore indirectly related to glottic closure and thus to the restoration of proprioceptive perception of subglottic pressure. Furthermore, the observation of the correlation of the MeAF with the connectivity at rest in this patient has allowed to highlight different points. The resting-state connectivity between voice motor/sensory nuclei bilaterally was more important when the MeAF was high; therefore, when the glottic air leak was important, when the vocal cord was paralyzed. The resting-state connectivity between the left brainstem nuclei and the motor, premotor, thalamic and cerebellar regions was also more important when the MeAF was high. This increased connectivity could reflect an attempt to compensate from the healthy side. On the paralyzed vocal fold side (right), direction of the correlations varied.

Although both of these elements support the hypothesis that restoring proprioceptive feedback could be beneficial, the effect of restoring auditory feedback cannot be isolated in the present study. Indeed, the injection generates both a restoration of the subglottic pressure and an improvement of the vocal quality. The patient could therefore hear herself again with a more stable and powerful voice after the intervention. Auditory feedback may also play a role. The study of Smith et al. (2020), which investigated the effect of disrupting auditory feedback and proprioceptive feedback on the laryngeal compensatory response, concluded that when both sources of feedback were available and the information received by these two perceptual systems was congruent, the compensatory response was greater. Although this study was not conducted in patients with voice disorders, the presence of auditory feedback may potentiate the effect of proprioceptive feedback detected in the voice-related nuclei in the brainstem.

Qualitatively, an important observation was that the patient reported being able to clearly identify the moment her “normal” voice started to return. This occurred 3 months after the injection (between time 2 and time 3). She then commented that her voice was clearer already in the morning, that there was no longer a hitch, and that the voice always came out as she expected. This patient was therefore particularly attentive to her senses, whether they were sensory or audible.

All the observations above invited questions about the conditions that might foster the reinnervation process. Some of these have been described previously. First, the severity of the nerve injury as well as the distance between the damage and the vocal muscle impact the possibilities of nervous recovery (Mau et al., 2017). Neurotmesis or axonotmesis are less likely to result in reinnervation to restore mobility than neurapraxia. Indeed, the more the nerve fibers are damaged, the more the risk of having a synkinetic reinnervation is important (Mau et al., 2017). Although it is not easy to have a clear indication of these parameters, different aspects can provide an indication. LEMG shows a good positive predictive value for screening patients with poor recovery prognosis (Wang et al., 2015) but can also inform on the topology of the injury. When the nerve signal is intact in the crico-thyroid muscle, the damage is located further along the vagus nerve, at the level of the recurrent laryngeal nerve. In addition, the etiology is also informative. Indeed, recoveries are more frequent in idiopathic or infectious UVFP (Sulica, 2008). A hypothesis on the severity can also be formulated according to the type of surgery performed. Second, the activation observed of the brainstem voice-related nuclei leads us to consider the need for sensory innervation to remain available for proprioceptive feedback to be transmitted. Regarding the vocal folds contact, this transmission is ensured by the superior laryngeal nerve. Regarding the subglottic pressure, it could be ensured by some sensory anastomosis with the recurrent laryngeal nerve. The impairment or not of these nerves could therefore be a determining factor. Third, in order for proprioceptive feedback to be effective, glottic closure must be complete during phonation. This condition depends on the position and the possible amyotrophy of the paralyzed vocal fold. The vocal fold could be paralyzed in the paramedian position but, when this is not the case, the injection could carry out this function. If this procedure is required, it is preferably performed as early as possible after the nerve injury. Indeed, it has been described that the chances of nerve recovery are higher just after the injury and decrease significantly with time to be minimal from 9 months (Mau, 2019). This would be a fourth condition.

This study presents several points of improvement for future studies. First, regarding the experimental design, it would be beneficial to record the patients with a microphone during the task and have them hear themselves live with auditory feedback from the microphone. This would permit the use of the prerecorded [a:] only in the AUDITION task and ensure that the task was performed as instructed. In addition, a choice was made regarding the analyzed behavioral measures. This choice was justified based on the principle of having a representative measure of the different parameters of the voice: acoustic, aerodynamic, vocal fold mobility and peripheral innervation. These last two parameters were analyzed with qualitative scales previously reported. Other teams may consider it more relevant to measure quantitative parameters for these two aspects. Concerning the fMRI analyses performed, some of them were based on regions of interest defined in a previous literature review. They were representative of the regions reported to be activated by a sustained vowel phonation task but were not always bilateral. It was therefore not always possible to distinguish between compensation of the healthy vocal fold and reinnervation attempts in the paralyzed vocal fold. Bilaterality could only be commented on at the level of the brainstem voice-related nuclei and of the laryngeal motor cortex for which the clusters were systematically bilateral as also for the qualitative analyses in the whole brain. In the future, the contralateral coordinates should always be included for each ROI in the investigated regions. Finally, given the intrinsic heterogeneity of this voice disorder, we recommend privileging case series analyses.

More hypotheses than answers were addressed in this study. Given the small number of profiles analyzed, it was only possible to describe and share the methodology and our observations. Only one patient received injection, we formulated several conditions for the replicability of our observations with this patient and we hope that other patients with similar profiles will support our assumptions. Furthermore, we have to be aware of the possibility that P3 could have recovered spontaneously without the injection. Our assumptions are therefore not intended to encourage a more systematic early injection but to encourage understanding of whether and how early injection augmentation might influence the nerve recovery process. We hope that both the experimental design and the results discussed will encourage further research on this exciting and crucial issue in order to develop and provide the best possible intervention for each UVFP patient.

## Conclusion

This multiple-cases longitudinal study highlighted several points. In our three patients, an hyperactivation was observed at time 1 for the sustained vowel phonation task. This hyperactivation was related to an increase in resting-state connectivity between phonatory regions of interest. Moreover, two patients recovered vocal fold mobility but the brain plasticity related to this nerve recovery did not follow a similar trend. We did not identify a single pattern of brain plasticity related to recovery; this could depend on whether the recovery is spontaneous or supported by an early intervention. Finally, for the patient who received an augmentation injection in the paralyzed vocal fold, we subsequently observed a bilateral activation of the voice-related nuclei in the brainstem. This last observation, as well as the fact that, for this same patient, the resting-state connectivity between the voice motor/sensory brainstem nuclei and other brain ROI correlated with the MeAF, support the hypothesis that promoting the restoration of proprioceptive feedback enhances the neural recovery process. These observations should be investigated in future research. We have therefore provided a list of conditions in which we believe that a similar observation at the level of the voice-related nuclei in the brainstem could be expected. The qualitative results obtained with our multiple-cases report provides further insight about the process at play in vocal recovery and pave the way for more efficient treatments for patients dealing with UVFP.

## Data availability statement

The datasets presented in this article are not readily available because this is a multiple-case study and the patients included did not give their consent for the data to be used by other research teams. Requests to access the datasets should be directed to MD, marie.dedry@uclouvain.be.

## Ethics statement

The studies involving human participants were reviewed and approved by the Ethics Committee of the University Hospital of Saint-Luc (number: B403201837695). The patients/participants provided their written informed consent to participate in this study.

## Author contributions

MD, LD, VV, AS, YM, and GD contributed to the conception and design of the study. MD, LD, VV, DB, and GD participated in the data collection. MD and LD performed the analysis. MD, LD, AS, YM, and GD contributed to the interpretation of the results. MD wrote the first draft of the manuscript. LD wrote sections of the manuscript. All authors contributed to the manuscript revision, read, and approved the submitted version.

## Footnotes

1
http://www.pstnet.com

2
https://www.nordicneurolab.com
