# Supplementary material for: Brain adaptation following various unilateral vocal fold paralysis treatments: A magnetic resonance imaging based longitudinal case series
Source: Front Neurosci. 2022 Oct 5;16:947390. doi: 10.3389/fnins.2022.947390 (PMC9580273; doi:10.3389/fnins.2022.947390)
Supplement: Supplementary file 1 [file Data_Sheet_1.pdf]

Supplementary material

Table 1

Collection procedure of acoustic and aerodynamic voice measures

All voice samples were collected in a quiet room and recorded with a stand microphone placed 5 cm from the patient's mouth and positioned at an angle so as not to face the airflow.

	Voice material	Software	Method
Acoustic measures			
Intensity range (dB)	[ɔ]	VRP	
Mean F0 (Hz)	[a:], 3 sec.	MDVP	
F0 range (Hz)	[ɔ]	VRP	
Jitter	[a:], 3 sec.	MDVP	
Shimmer	[a:], 3 sec.	MDVP	
NHR	[a:], 3 sec.	MDVP	
CPPS	[a:], 3 sec.	Praat	Praat script[1]
Aerodynamic measures			
MPT	[a:] as long as possible	/	3 times, longest is retained
VC	Expiratory airflow	PAS 6600	3 times, highest is retained
PQ	/	/	MPT/VC
MeAF	"We were away a year ago."	PAS 6600	
ESGP	/pa, pa, pa, pa, pa/	PAS 6600	

Notes. "F0" means fundamental frequency, "NHR" means noise-to-harmonic ratio, "CPPS" means smoothed cepstral peak prominence, "MPT" means maximum phonation time, "MeAF" means mean air flow, "VC" means vital capacity, "PQ" means phonatory quotient, "ESGP" means estimated subglottic pressure. "MDVP" is the Multi-Dimensional Voice Program (MDVP, Computerized Speech Lab (CSL™), KayPentax, Lincoln Park, NJ). "VRP" is the Voice Range Profile (VRP, Computerized Speech Lab (CSL™), KayPentax, Lincoln Park, NJ). Praat is a free software for sound analysis, manipulation and annotation (Praat, Boersma & Weenink, Phonetic Science, Amsterdam, Netherlands). PAS 6600 is the Phonatory Aerodynamic System Model 6600 (KayPentax, Licoln Park, NJ). [1]Praat script used to calculate the cepstral peak prominence measure as defined by Hillenbrand et al. (1994) was based on https://www.fon.hum.uva.nl/praat/manual/PowerCepstrum__Get_peak_prominence___.html

Table 2

Regions of interest (ROI) in MNI coordinates

X	Y	Z	ROI Name	RS analyses (mm³)
-50	-9	31	BA4_PreG_Cl_L1	515
48	-7	31	BA4_PreG_Cl_R1	486
-45	-13	38	BA4_PreG_Cl_L2	515
44	-12	38	BA4_PreG_R2	515
-18	-30	58	BA4_PreG_L3	515
19	-29	62	BA4_PreG_Cl_R3	500
-60	-4	36	BA6_PreG_L1	479
64	-5	28	BA6_PreG_R1	391
50	-7	38	BA6_PreG_Cl_R2	515
-49	-7	47	*BA6_PreG_L3*	512
-55	5	22	*BA6_PreG_L4*	233
61	6	17	*BA6_PreG_R4*	440
-56	-7	20	BA43_PreG_Cl_L1	467
58	-26	24	*BA43_ParOperc_R2*	398
-6	-2	68	BA6_SupFrontG_L1	511
1	0	66	BA6_SupFrontG_R1	428
-4	-4	62	*BA6_ParacentralLobule_L1*	515
8	-4	60	*BA6_ParacentralLobule_R2*	297
48	11	6	BA44_InfFrontG_oper_Cl_R1	234
41	19	6	*BA44_InfFrontG_oper_R2*	435

-33	15	-10	OP47_InfFrontF_orbit_L1	
-51	49	-7	OP47_InfFrontF_orbit_L2	
32	36	0	OP47_InfFrontF_orbit_R3	
40	54	-3	OP47_InfFrontF_orbit_R4	68
-26	28	50	*BA8_SupFrontG_Cl_L1*	
25	30	47	*BA8_SupFrontG_R1*	
68	-6	16	*BA1_PreG_R1*	257
62	-15	2	BA42_PlanTemp_Cl_R1	513
-56	-32	19	*BA42_PlanTemp_L2*	40
56	-26	14	*BA42_PlanTemp_R2*	426
-57	-37	12	BA22_SupTempG_Cl_L1	296
-61	-14	9	BA41_AntTransvTempG_L1	203
-42	-24	8	BA41_AntTransvTempG_L2	7
51	-18	3	BA41_AntTransvTempG_Cl_R3	16
58	-13	6	*BA41_AntTransvTempG_R4*	479
-60	-6	8	BA42_AntTransvTempG_Cl_L1	288
61	-5	6	BA42_AntTransvTempG_Cl_R1	513
-42	-77	24	*BA37_MidTempG_L1*	
45	-69	18	*BA37_MidTempG_R1*	27
41	-78	2	*BA37_MidTempG_R2*	
51	-39	7	*BA21_MidTempG_R1*	317
-47	-37	32	BA40_ParietalOper_Cl_L1	515
50	-38	32	BA40_ParietalOper_Cl_R1	47

47	-31	27	BA40_ParietalOper_R2	
29	-40	37	*BA7_SupramarginalG_R1*	38
-6	12	38	*BA24_CingulateG_L1*	447
10	8	35	*BA24_CingulateG_Cl_R1*	190
10	14	34	*BA32_CingulateCortex_R1*	294
-2	-54	23	*IsthmCingulateG_L2*	
8	-54	21	*IsthmCingulateG_R2*	
-17	-68	14	*PostCingulateG_L3*	470
11	-66	13	*PostCingulateG_R3*	219
-30	0	16	Insula_L1	
-38	5	0	Insula_L2	178
41	-2	15	Insula_R3	5
-23	-4	6	Putamen_L1	
22	3	6	Putamen_R1	251
24	-4	-2	Putamen_R2	88
32	-8	12	Putamen_R3	109
26	-2	-8	*PiriformCortex_R1*	
-26	-8	-6	*AmygdaloidIsland_L1*	
22	-10	-4	*IntGlobusPallidus_R1*	18
-14	-20	0	Thalamus_L1	
14	-18	4	Thalamus_R1	112
14	-8	16	Thalamus_R2	
-26	-58	-28	Cerebellum_Cl_VI_L1	397

20	-60	-20	Cerebellum_VI_R1	356
-12	-64	-16	Cerebellum_VI_L2	494
-21	-64	-21	Cerebellum_VI_L3	501
3	-88	-34	Cerebellum_CrusII_R4	
33	-58	-23	Cerebellum_VI_R5	209
5	-40	-52	*Brainstem_MotSens_R*	12
-5	-40	-52	*Brainstem_ MotSens_L*	12

Notes. In the "ROI Name" column, the names written straight are the regions of interest (ROI) reported in the previous literature review paper (Dedry et al., 2022), those in italics have been added. The column "Included in RS analyses" specifies, in mm^3, the size of the 55 ROI that were included in resting-state analyses.
